# An improved chaotic fruit fly optimization based on a mutation strategy for simultaneous feature selection and parameter optimization for SVM and its applications

**DOI:** 10.1371/journal.pone.0173516

**Published:** 2017-04-03

**Authors:** Fei Ye, Xin Yuan Lou, Lin Fu Sun

**Affiliations:** School of Information Science and Technology, Southwest Jiaotong University, ChengDu, China; Beihang University, CHINA

## Abstract

This paper proposes a new support vector machine (SVM) optimization scheme based on an improved chaotic fly optimization algorithm (FOA) with a mutation strategy to simultaneously perform parameter setting turning for the SVM and feature selection. In the improved FOA, the chaotic particle initializes the fruit fly swarm location and replaces the expression of distance for the fruit fly to find the food source. However, the proposed mutation strategy uses two distinct generative mechanisms for new food sources at the osphresis phase, allowing the algorithm procedure to search for the optimal solution in both the whole solution space and within the local solution space containing the fruit fly swarm location. In an evaluation based on a group of ten benchmark problems, the proposed algorithm’s performance is compared with that of other well-known algorithms, and the results support the superiority of the proposed algorithm. Moreover, this algorithm is successfully applied in a SVM to perform both parameter setting turning for the SVM and feature selection to solve real-world classification problems. This method is called chaotic fruit fly optimization algorithm (CIFOA)-SVM and has been shown to be a more robust and effective optimization method than other well-known methods, particularly in terms of solving the medical diagnosis problem and the credit card problem.

## 1. Introduction

In many real-world classification tasks, reducing the dimensionality of data is an essential step before classifying the data. The general approach for reducing the dimensionality of data involves feature selection techniques that aim to select the most relevant features based on certain predefined filter criteria. Based on their dimensionality-reducing characteristics, feature selection techniques have been widely applied in many pattern recognition tasks and machine learning fields.

Many classification tasks require sophisticated systems or tools to sort the unlabeled samples into the correct class. Machine learning classification algorithms have thus been widely applied to solve classification task problems because of the advantages that can be derived from their inherent characteristics. These classification algorithms include support vector machine (SVM) [1], Decision Trees (DT) [2], K-Nearest Neighbor (K-NN) [3], Naïve Bayes Networks (NB) [4], and Artificial Neural Networks (ANN) [5]. One of the most popular and frequently used classification algorithm is SVM, which is based on the structural risk minimization principle and Vapnik-Chervonenkis theory [6]. Many studies have demonstrated that SVM has powerful generalization capability and better classification performance than other well-known classification algorithms [7–8]. However, traditional SVM has its own weaknesses and strengths. In recent years, to overcome the drawbacks and improve the classification performance of standard SVM, several optimized models based on the original SVM have been proposed, including V-SVM [9], least squares SVM [10], NPSVM [11], Twin SVM [12], and nearly isotonic SVM [13]. In many classification tasks, SVM generally learns a nonlinear and high-dimensional set of samples that contains much irrelevant attribute information and noise data, which can lower the classification performance and computing efficiency of the SVM classifier. Feature selection techniques have been employed to select the optimal feature subset for the SVM model to enhance its generalization ability and to preserve the computational cost of the SVM classifier. Moreover, parameter setting turning for the SVM model also plays an important role in affecting the performance of the classification. Thus, many previous studies have simultaneously addressed the parameter setting and performed feature subset selection for the SVM classifier [14–15]. To optimize SVM’s parameter setting, the key parameters must be optimized, such as the penalty parameter C, which controls the trade-off between model complexity and fitting error minimization, and the hyperplane parameter *γ*, which is the kernel bandwidth of the radial basis function (RBF), which should be properly optimized before performing classification tasks.

To optimize SVM, several swarm intelligent algorithms have been proposed to address the parameter settings and to select an optimal feature subset for the SVM classifier, such as genetic algorithms (GA) [16], particle swarm optimization algorithms (PSO) algorithms [17], artificial immune algorithms (AIA) [18], and ant colony algorithms [19]. Although these swarm intelligent algorithms have been proposed to address parameter settings and to select a proper feature subset for the SVM classifier, they are complicated to implement and difficult to understand. In recent years, the fruit fly optimization algorithm (FOA), a novel member of this group of swarm intelligent algorithms, was first proposed by Pan W et al. [20] in 2012; FOA imitates the foraging behavior of fruit flies. The main outstanding features of FOA are that it is easy to understand, contains a simple searching procedure, and is simple to implement. Due to its good performance and excellent properties, FOA has been widely employed in many real-world classification tasks and in the SVM optimization field. For example, Lei X et al. [21] used the FOA in combination with gene expression profiles to solve problems involving the identification of dynamic protein complexes. The experimental results showed that this method is more effective at detecting protein complexes than the other well-known methods. To construct an optimal stand-alone hybrid photovoltaic (PV)-wind-diesel-battery system [22], a new and improved FOA that used a multi-objective optimization method was proposed to optimize this system. The experimental results showed the feasibility of the stand-alone hybrid PV-wind-diesel-battery system optimized by this method for Dongao Island. A modified FOA called novel 3D-FOA was proposed by Lin W et al. [23] that aimed to improve the original FOA in terms of several nonlinear functions, and the results showed its superiority. Mousavi S et al. [24] proposed an improved FOA, namely, CIFOA, which aims to solve the homogeneous fuzzy series–parallel redundancy allocation problem under discount strategies. To solve the hybrid flow-shop rescheduling problem with flexible processing time in steelmaking casting systems [25], a hybrid method using the FOA and two decoding heuristics called HFOA was proposed and has successfully solved flow-shop rescheduling problems. To optimize continuous function problems, Wang L et al. [26] proposed an improved FOA that uses a new mutation parameter and level probability policy. In this improved algorithm, the mutation parameter and level probability policy are used to balance population stability and diversity. To optimize twin support vector machine (TWSVM), at least two parameters generally must be considered. Ding S et al. [27] used FOA for parameter setting turning of TWSVM, and the experimental results showed that using FOA to optimize TWSVM can yield better classification performance than SVM. To solve the medical data classification problem, [28] used FOA to optimize SVM by determining an optimal parameter setting of the SVM model; compared with other well-known methods, the results of the constructed experiments of this method showed that the optimized SVM model with FOA is a powerful tool for medical data classification. For community detection methods, which are generally based on one evolutionary algorithm, a novel multi-swarm FOA was proposed by Liu Q et al. [29], namely, CDMFOA, and the experimental results showed that this method can effectively solve the detection community structure. For the nonlinear and non-stationary traits of rotating machinery vibration, the SVM classifier optimized by FOA has been used [30], and the experimental results showed that using FOA in combination with SVM (FOA-SVM) can yield better performance with respect to rolling bearing diagnosis. Some control systems have certain essential parameters that require proper determination [31]. A new hybrid method using GA in combination with FOA has been proposed to perform parameter tuning of control systems. In this method, FOA is employed to perform parameter setting turning of the controller system and GA is used to select the controller structure. Si L et al. [32] used an improved FOA in combination with the least squares support vector machine (LSSVM) to solve the identification problem of Shearer Cutting Patterns and constructed experiments to compare with PSO-LSSVM, GA-LSSVM and FOA-LSSVM; these experiments indicated that the proposed improved fruit fly optimization algorithm (IFOA)-LSSVM outperformed other methods. FOA has also been applied in traffic flow forecasting. A method using FOA to optimize the LSSVM has been proposed to improve the accuracy of traffic flow forecasting [33], and the experimental results showed that this method outperformed the LSSVM model, the RBF neural network (RBFNN), and LSSVM-PSO. To overcome the disadvantages of traditional FOA, Wu L et al. [34] proposed a cloud mode based on FOA, namely, CMFOA, which uses an adaptive parameter strategy to enhance the global search ability in the first stage, and performed experiments using 33 benchmark functions, and the results revealed the superior performance of this method compared with that of other FOA variations. One of the most important drawbacks of the original FOA is that it is difficult to obtain optimal solutions in zero vicinity; thus, an improved algorithm based on the original FOA using differential evolution has been proposed [35], namely, DFOA, which modifies the representation of the smell concentration judgment value and replaces the stochastic search with a differential vector. The experimental results show the effectiveness of DFOA for finding optimal working conditions. Parameter estimation plays an important role in bidirectional inductive power transfer (BIPT) systems. To obtain a proper parameter setting for this system, an improved algorithm using chaotic PSO to enhance the original FOA has been proposed [36], namely, CFOA, and the experimental results showed that the 11 parameters of this system were determined properly. In certain real-world problems, such as joint replenishment problems (JRPs), FOA has also been applied. Wang L et al. [37] proposed an improved and effective algorithm based on the original FOA, namely, IFOA, which is used to solve joint replenishment problems and to optimize numerical functions. To determine product specifications, the melt index (MI) is one of the most important criterion. To forecast MI, an improved FOA using an adaptive mutation, namely, AM-FOA [38], was used to determine the punishment factor “γ” and the parameters of the Gaussian RBF kernel, and the experimental results showed that AM-FOA optimizing LSSVM is a functional method in MI prediction. FOA is generally suitable for optimizing continuous variables. To solve the discrete variable optimization problem, a binary FOA has been proposed to solve set covering problems (SCPs) [39]. Pan Q K et al. [40] proposed an improved algorithm based on the original FOA, namely, IFFO, which is used to solve continuous function optimization problems. The main novelty of this algorithm is that it uses a new parameter to control the search scope, and the experimental results showed that IFFO outperforms five state-of-the-art harmony search algorithms. To solve the semiconductor final testing scheduling problem (SFTSP), a novel algorithm based on the original FOA was proposed [41] called nFOA; multiple fruit fly swarms are employed in the evolution process to improve FOA’s parallel search ability. One typical discrete optimization problem is solving three-dimensional path planning. The IFOA has been proposed [42] for solving engineering problems. The constructed experiments have shown that IFOA is a powerful method that can solve discrete optimization problems with greater efficiency.

As the above related works of FOA show, FOA has become a powerful tool to effectively determine proper parameter settings for machine learning algorithms and to successfully solve complex multidimensional problems. However, traditional FAO has several drawbacks, such as the searching procedure becoming easily trapped in the local optimum, premature convergence, and the difficulty of addressing the discrete variable optimization issues. Several studies have proven that FOA is an efficient tool for parameter estimation of machine learning algorithms, such as the GRNN and SVM. Moreover, according to the related work of SVM parameter optimization, most of these optimization methods using FOA perform only parameter turning for the SVM classifier without simultaneously performing feature selection. Therefore, the above facts motivate us to propose a novel, intelligent framework using the proposed CIFOA and its improved mutation strategy, aiming to enhance the generalization ability and improve the classification performance of the SVM classifier by determining a proper parameter setting with an optimal feature subset simultaneously. In the proposed CIFOA, the chaotic PSO is proposed in combination with the mutation strategy to overcome the weaknesses of the original FOA and make the algorithm procedure reflect ergodicity, randomicity, and regularity. The proposed CIFOA optimizing SVM, namely, CIFOA-SVM, is an efficient framework that can be used to solve various real-world classification problems. The main novelty of the proposed intelligent framework is using chaotic PSO to solve the discrete variable optimization problem (determining an optimal feature subset). The proposed mutation strategy uses two different searching strategies to search for the local and global optimal solutions simultaneously, whereas a mutation parameter is proposed to allocate the individual in both the local searching strategy and global searching strategy. Concurrently, this study proposes a weighted fitness function to simultaneously address the trade-off between sensitivity and specificity classification accuracy and the number of selected features to maximize the classification performance of the proposed method. The efficiency and effectiveness of the proposed method have been examined in terms of classification accuracy, sensitivity, specificity, running time, and convergence curve with respect to two real-world classification problems: the medical diagnosis problem and the credit card problem. Five real-world datasets are introduced to evaluate the classification performance of various methods in solving real-world problems, and these datasets come from the UCI machine learning database repository. The experiment’s results indicate that the proposed method can determine a more appropriate SVM model parameter setting and obtain an optimal feature subset with much less running time than the GAFS and other intelligent methods. The main contributions of this study are as follows: (1) it develops an improved FOA based on chaotic particle optimization with a novel mutation strategy, and (2) it develops an improved FOA that is successfully applied to SVM to determine proper parameter settings with an optimal subset of features for real-world classification problems.

The remainder of this paper is organized as follows: in section 2, we provide the necessary background materials regarding SVM and FOA. The proposed improved FOA based on chaotic optimization techniques and mutation strategy is presented in section 3 in which we also provide several groups of tests on well-known continuous functions. The detailed experiments and in-depth comparisons of the proposed framework with other well-known methods are presented in section 4, in which we also include a discussion. Finally, the conclusion and recommendations for future research are summarized in section 5.

## 2. Background materials

### 2.1. A brief overview of support vector machines

In this subsection, we present a brief description of SVM. SVM was originally developed by Vapnik et al. [43] and is mainly used to solve classification problems. In subsequent years, SVM was applied to the multi-classification problem [44–45]. The main objective of SVM is to determine an optimal hyperplane that separates data of different classes on either side. The optimal hyperplane is determined by maximizing the interval between the support vectors of the closest positive and negative frontiers.

For now, we consider binary classification problems, i.e., -1 or 1, to represent one of two classes of a sample. When the label of i item of the samples is -1, then that i item of the samples belongs to the “positive class”; otherwise, the i item of the samples belongs to the “negative class”. Let *D*_*i*_ = {*X*_1,_*X*_2_.…,*X*_*n*_,*Y*_*i*_),*i* = 1,2..*n*,*Y*_*i*_ ∈ {−1,1}, where *D*_*i*_ represents i item of the samples. *Y*_*i*_ is the label of i item of the samples. To separate the instances into two categories, we use the function *F*(*X*) = **W**^**T**^**X** + *b*, where **W** is a coefficient vector that is used to normalize the hyperplane. For the linearly separable case, an optimal separating margin can be determined by solving the following equation:

$$\begin{array}{l} \underset{w,b,\varepsilon }{\mathrm{MIN}}\frac{1}{2}W^{T}W+C\sum_{i=1}^{n}\varepsilon_{i}\\ \mathrm{subject}\;\mathrm{to}:\\ Y_{i}(W^{T}X_{i}+b)\geq 1-\varepsilon_{i},\varepsilon_{i}\geq 0 \end{array}$$ (1)

To solve the above equation, a dual Lagrangian equation with multipliers *α*_*i*_(*i* = 1,2,…*n*) is introduced. The detailed Lagrangian equation can be expressed as follows:

$$\begin{array}{l} \underset{\alpha }{\mathrm{MAX}}\mathrm{La}(\alpha )=\sum_{i=1}^{n}\alpha_{i}-\frac{1}{2}\sum_{i,j=1}^{n}\alpha_{i}\alpha_{j}Y_{i}Y_{j}X_{i}X_{j}\\ \mathrm{subject}\;\mathrm{to}:\\ 0\leq \alpha_{i}\leq C,i=1,2,.\ldots ,n,\sum_{i=1}^{n}\alpha_{i}Y_{i}=0 \end{array}$$ (2)

To construct the optimal hyperplane, a Lagrangian equation La(*α*) must be maximized with a positive multiplier *α*_*i*_ under the conditions of $\sum_{i=1}^{n}\alpha_{i}Y_{i}=0$ and *α*_*i*_ ≥ 0. The solution *α*_*i*_ can be solved by addressing the parameter w^*^ and b^*^ of the optimal hyperplane. Thus, we can introduce an optimal equation for solving this case.

$$\begin{array}{l} f(X,\alpha^{*},b^{*})=\sum_{i=1}^{n}Y_{i}\alpha_{i}^{*}<X_{i},X_{j}>+b^{*}\\ =\sum_{i\in sv}^{sv}Y_{i}\alpha_{i}^{*}<X_{i},X_{j}>+b^{*} \end{array}$$ (3)

From the above equation, the Lagrangian multiplier *α*_*i*_ = 0 means that its corresponding training vector is the closest to the margin of the optimal hyperplane and is also called a support vector. The main SVM characteristic is clearly expressed, i.e., constructing the optimal hyperplane depends on only a small subset of the training dataset without all the training dataset.

To address the nonlinear case, the linear equation can also be modified to address the nonlinear data. For now, a general ideal is given here, i.e., we use a kernel function to map the original input spaces into a higher-dimensional feature space. The original input data can be linearly separated using the kernel function to calculate the inner product in the feature space.

The kernel function can be expressed as follows:

$$K\left(X_{i},X_{j}\right)=\Phi {\left(X_{i}\right)}^{\text{T}}\cdot \Phi \left(X_{i}\right)$$ (4)

By using the kernel function, the original linear generalized equation can be modified to represent the nonlinear dual Lagrangian La(*α*).

$$\begin{array}{l} \mathrm{La}\left(\alpha \right)=\sum_{i=1}^{n}\alpha_{i}-\frac{1}{2}\sum_{i,j=1}^{n}\alpha_{i}\alpha_{j}Y_{i}Y_{j}K\left(X_{i},X_{j}\right)\\ subject\;to:\\ 0\leq \alpha_{i}\leq C,i=1,2,\ldots ..,n,\sum_{i=1}^{n}\alpha_{i}Y_{j}=0 \end{array}$$ (5)

To solve the above optimization model, a method that solves the Lagrangian equation in the separable case can also be used to solve this optimization model.

$$f(X,\alpha^{*},b^{*})=\sum_{i=1}^{n}Y_{i}\alpha_{i}^{*}K\left(X_{i},X_{j}\right)+b^{*}$$ (6)

There are several general kernel functions, such as the radial basic function (RBF), polynomial, linear kernel function and sigmoid kernel function. Table 1 displays the detailed calculation of the four kernel functions.

**Table 1:** The expression of several classes of kernels.

Name	Caculation
Linear	*K*(*x*,*y*) = *x* × *y*
Polynomial	*K*(*x*,*y*) = (*ax* × *y* + *b*)^*d*^
RBF	*K*(*x*,*y*) = exp(−|*x*−*y*|^2^)/*δ*^2^
Sigmoid	*K*(*x*,*y*) = 1/(1 + exp(−|*x*−*y*|))

Where *d* is the polynomial order and *γ* is a parameter predefined by the user, which is used to control the graph’s width of the Gaussian kernel.

### 2.2. A brief overview of the basic fruit fly swarm optimization algorithm

**Fig 1:**
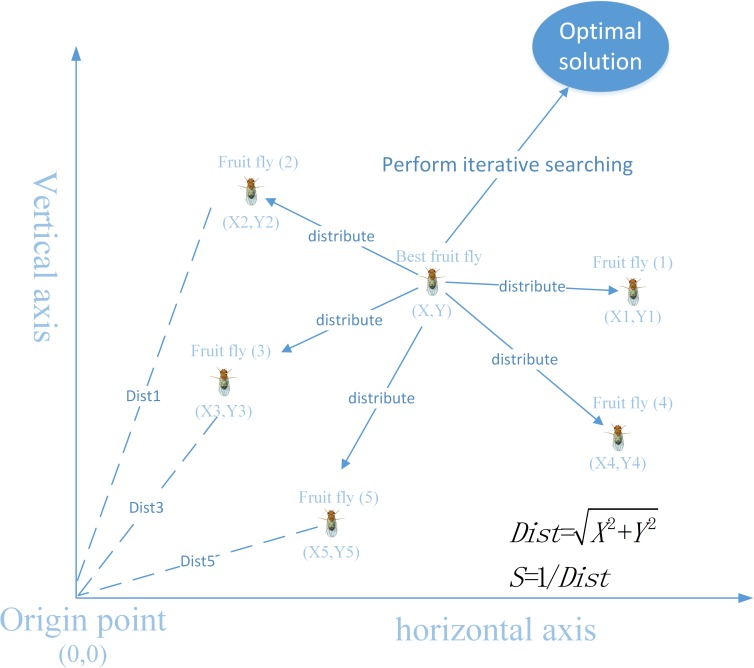
The food searching process of a fruit fly swarm.

FOA was developed by Pan W T et al. [46]. The main characteristics of FOA are to search for food source by visual sense and sensitive olfactory. The specific procedure a fruit fly swarm employs in searching for food is shown in Fig 1. More specifically, a fruit fly can still find food at a distance of 70 km from the food source. The main merits of FOA are that it is efficient, simple and easy to implement. FOA is generally suitable for solving continuous variables optimization problems. To solve discrete parameter optimization problems, the binary fruit fly optimization algorithm (bFOA) was proposed by Wang L et al. [47]. In bFOA, the MKP problem was represented by a binary string and three search procedures were used to perform an evolutionary search to obtain the local best solution and global best solution. Because of the nature of FOA, it works better with continuous variables optimization problems. Although FAO has better search ability than other intelligent algorithms, there are certain drawbacks of the original FOA that cannot effectively solve complex problems in the real world. Thus, some improved or modified algorithms have been proposed to overcome these drawbacks. For example, an improved algorithm using the original FOA in combination with chaotic PSO has been proposed [48], namely, CFOA, which was compared with other intelligent algorithms to solve ten well-known benchmark problems, and the results showed that CFOA outperforms the other algorithms as measured by various performance criteria. A bimodal optimization algorithm based on the original FOA and cloud model learning has been proposed [49], namely, BCMFOA, which uses an adaptive parameter update strategy and a cloud generator to adaptively adjust the search range of fruit flies. To solve the drawback of the original FOA in multidimensional complicated optimization problems, a novel FOA algorithm using the multi-swarm fruit fly concept has been proposed [50], namely, MFOA. In comparison with the original FOA, the constructed experiments of MFO showed that MFOA obtains a significant outcome for several benchmark functions.

The original FOA can be divided into several steps. In this section, we attempt to express the detailed procedure of each step in FOA; then, we present the improved FOA in section 3.1. The detailed procedure of the original FOA is described as follows:

Step 1. FOA parameter initialization: to initialize the parameter setting for FOA, several necessary parameters must be considered. The parts of these parameters are the same as those of other evolutionary algorithms (EAs), such as population size and the maximum iteration number. The remaining parts of these parameters are the upper bound with the lower bound of the random flight distance range and the initial fruit fly swarm location (*X*_*axis*_,*Y*_*axis*_). The initial location (*X*_*axis*_,*Y*_*axis*_) can be initialized by solving the following equations:

$$X_{axis}=1+rand(),$$ (7)

$$Y_{axis}=1+rand(),$$ (8)

where *rand*() is used to generate a random value in the interval [0, 1].

Step 2. Initializing populations for FOA: initializing the population for FOA employs a random strategy with the obtained initial fruit fly swarm location to generate a random location (*X*_*i*_,*Y*_*i*_) for each fruit fly. (*X*_*i*_,*Y*_*i*_) represents the location of the *i-th* fruit fly and is obtained as follows:

$$X_{i}=X_{axis}\pm rand(),$$ (9)

$$Y_{i}=Y_{axis}\pm rand(),$$ (10)

Step 3. Evaluating the population for FOA: to evaluate the population and determine the best fruit fly, the distance from each fruit fly location to the food location can be used to calculate the smell concentration judgment value. *Distance*_*i*_ represents the distance of the *i-th* fruit fly to the food location, which can be calculated by solving Eq (11). The smell concentration judgment value *S*_*i*_ is obtained by solving Eq (12) as follows:

$$\mathrm{Distance}\left(i\right)=\sqrt{X_{i}^{2}+Y_{i}^{2}}$$ (11)

$$S_{i}=1/\mathrm{Distance}\left(i\right)$$ (12)

Step 4. Converting the concentration value to fitness value: from the above step, the smell concentration judgment value of each fruit fly has been obtained. Converting the smell concentration judgment value to the fitness value is a convenient way to evaluate the importance of each fruit fly in populations.

$$Smell_{i}=\mathrm{FitnessFunction}\left(S_{i}\right)$$ (13)

Step 5. Selecting the maximal smell concentration: to find the food source, the maximal smell concentration is used as a guide for the searching procedure. *bestSmellIndex* represents the corresponding index of the maximal smell concentration among the fruit fly swarm.

bestSmellIndex=SelectionMax(Smell) (14)

Step 6. Updating the maximal smell concentration: this step updates the maximal smell concentration value and location (*X*_*axis*_,*Y*_*axis*_) based on the determination of *bestSmellIndex*, which means that all fruit flies modify their own location to move toward the direction of the maximal smell concentration.

$$X_{axis}=X\left(bestSmellIndex\right)$$ (15)

$$Y_{axis}=Y\left(bestSmellIndex\right)$$ (16)

Step 7. Checking the termination conditions: this step compares the current maximal smell concentration value with the previous maximal smell concentration value. If the current maximal concentration is no longer superior to the previous one, then the termination conditions are satisfied and the iterative procedure is stopped. Moreover, the maximum iteration number is needed to avoid unnecessary computational costs.

## 3. The proposed methodology

In this section, we present a novel and efficient FOA based on mutation strategy and chaotic PSO. The detailed mutation strategy is described in section 3.1. In section 3.2, we provide the detailed algorithm procedure of the CIFOA and its pseudo-code.

### 3.1. Mutation strategy and chaos particle optimization

In a traditional intelligent algorithm, the algorithm’s performance depends on its preset parameter setting and is easily trapped in a local optimum. To further avoid premature convergence and enhance the algorithm’s search ability, the chaos concept has been introduced [48, 51, 52, 53, 54, 55]. Moreover, many algorithms based on the chaos concept were proposed to solve the medical diagnosis problem [55], yielding excellent outcomes. Chaos is characterized as ergodic, random, and regular [56–58]. Numerous studies have shown that random-based optimization algorithms perform better when using non-standard distributions (i.e., Gaussian or uniform distributions). Additionally, the properties of ergodicity and non-repetition of the chaos technique can force an algorithm to perform overall searches at higher speeds. These are the main reasons to employ the chaotic technique used in the proposed algorithm.

Although traditional FOA can achieve considerable results in terms of search efficiency and running time in various fields, FOA’s searching performance depends exclusively on its fruit fly swarm location, which can easily lead the procedure of the FOA to fall into the trap of local optima. Thus, to address this problem and improve FOA’s searching ability, we introduce the chaotic PSO in combination with the proposed mutation strategies to be used in FOA. The mutation strategy is proposed to generate two different osphresis forging strategies for simultaneously searching for the local optimum and the global optimum. One of these strategies is the global searching stage, which replaces the random method that fruit flies employ to find food sources. Instead, new food sources are generated by Eq (17), as follows:

$$\begin{array}{l} X_{ij}=minX_{j}+\left(maxX_{j}-minX_{j}\right)\cdot C_{ij},j=1,2,..,d,\\ i=1,..,n \end{array}$$ (17)

From the above equation, *X*_*ij*_ is a newly generated food source in the range [*minX*_*j*_,*maxX*_*j*_]. *n* is population size and *d* is the number of decision variables. *C*_*ij*_ is the *i-th* column of the *j-th* row of the chaos set, which is calculated as follows:

$$\begin{array}{l} \text{Normalized}\left(X_{ij}\right)=\left(X_{ij}-minX_{j}\right)/\left(maxX_{j}-minX_{j}\right)\\ C_{0j}=\mathrm{Normalized}\left(X_{axis}^{j}\right),j=1,2,\ldots \ldots ,d\\ C_{ij}=\mathrm{Logistic}\left(C_{i-1j}\right),i=2,3,\ldots .,n \end{array}$$ (18)

From the above equation, Normalized() is employed to transform the fruit fly swarm location in the range [0, 1]. *C*_0*j*_ is the *j-th* dimension of the initial chaos vector, which is given by $\text{Normalized}\left(X_{axis}^{j}\right)$. Logistic() is a logistic chaos mapping that is defined by Eq (19). Then, using an iteration of the logistic chaos mapping with an initial chaos vector, a set of chaos vectors *C*_1_,*C*_2_,.….…*C*_1_ is generated.

$$x_{i+1}=ax_{i}\left(1-x_{i}\right),a=4$$ (19)

The second osphresis forging strategy is the local searching stage, which generates new food sources around the fruit fly swarm. In this stage, the osphresis parameter *μ* is proposed to help the algorithm control the range of newly generated food sources. The new food source *X*_*ij*_ is calculated as follows:

$$X_{ij}=X_{axis}^{j}\pm C_{ij}\cdot rand()\cdot \mu ,j=1,2,..,d$$ (20)

Note that the osphresis parameter *μ* plays an important role in the local searching ability of CIFOA and should be set properly. According to our previous experimental results, the osphresis parameter *μ* can be determined as follows:

μ=(upper_bound−lower_bound)/population_size (21)

From the above equation, the *upper_bound* and *lower_bound* are used to form a domain of the parameter.

To simultaneously perform the two stages in an iterative procedure, the mutation probability rate *mr* is introduced to allow many individuals to use the global searching stage, while for the remaining population uses the local searching stage. The mutation probability *mr* is generally set to 0.8; a specific procedure of the mutation probability rate *mr* used is described as follows:

$$\begin{array}{l} \text{if}\;rand()\leq mr\;\text{Then}\\ \;\text{Perform}\;\text{global}\;\text{searching}\;\text{stage}\\ \text{Else}\\ \;\text{Perform}\;\text{local}\;\text{searching}\;\text{stage} \end{array},$$ (22)

where *rand*() is a randomly generated value in the interval [0, 1].

### 3.2 The procedure of CIFOA

According to the nature of the proposed CIFOA, the full procedures of the proposed algorithm can be divided into seven steps, and each step is described in detail as follows:

Step 1. Parameters and chaos particle initialization: to gain an appropriate initial fruit fly swarm location and to maintain the diverse population distribution, the chaotic optimization technique is suitable for initializing the fruit fly swarm location and generating other fruit flies’ locations. Moreover, the population size, maximum number of iterations, mutation probability *mr*, and osphresis parameter *μ* must be initialized.

(1) Provide a chaos vector *C*_0_ = {*C*_01_,*C*_02_,..,*C*_0*n*_}, then use the random function to generate a random value in the range [0, 1] for each component *C*_0*i*_ of the chaos vector.

(2) Use the initial chaos vector *C*_0_ with the iteration procedure of the logistic chaos equation to generate a set of chaos vectors, *C*_1_,*C*_2_,*C*_3_,….,*C*_*n*_.

(3) To avoid the *i-th* item-value of the chaos vector being outside the bounds of the *i-th* parameter’s range, the data normalized method is used to transfer a chaos vector, *C*_*i*_, into the parameter’s range; the detailed transformation is thus described as follows:

$$C_{ij}^{'}=minX_{i}+(maxX_{i}-minX_{i})\cdot C_{ij},i=1,2,3,\ldots .m$$ (23)

where *maxX*_*i*_ and *minX*_*i*_ denote the upper and lower bounds of the *i-th* parameter’s range, respectively. $C_{ij}^{'}$ represents the value of the *j-th* dimension of the *i-th* fruit fly that has been converted into the range [*minX*_*i*_,*maxX*_*i*_].

Step 2. Determine the initial fruit fly swarm location: this step is mainly responsible for finding an appropriate location with a maximal smell concentration value as the initial fruit fly swarm location. First, calculate the smell concentration value of each fruit fly using a predefined evaluation function; then, compare the smell concentration value of each fruit fly to obtain an optimal fruit fly location as the initial fruit fly swarm location.

Step 3. Update the locations of fruit flies: this step employs the chaotic PSO with the initial fruit fly swarm location *X*_*axis*_ to update the location of the fruit flies. The detailed procedures are as follows:

(1) Consider the initial chaos vector *C*_0_ = {*C*_01_,*C*_02_,..,*C*_0*n*_}. The fruit fly swarm location *X*_*axis*_ is transferred to a scaled location that is used as the chaos vector *C*_0_, where each component must be converted into the range [0, 1] using the following equation:

$$C_{0}^{'}=\frac{C_{0i}-minX_{i}}{maxX_{i}-minX_{i}},i=1,2,\ldots ..m,$$ (24)

where *minX*_*i*_ and *maxX*_*i*_ are the lower and upper bounds of the parameter, respectively.

(2) The scaled vector $C_{0}^{'}$ is used as a chaos seed to generate a set of chaos vectors {*C*_1_,*C*_2_,……,*C*_*n*_} by iteration of the logistic chaos mapping.

(3) To update the location of the fruit fly, the proposed mutation parameter *mr* is employed to partition the population into two groups of fruit flies: one of the groups updates their locations through the global searching Eq (17), whereas the other groups update their locations using the local searching Eq (20).

Step 4. Calculate the smell concentration value: from the above step, the location of each fruit fly is obtained, given a parameter *X*_*i*_ that represents the distance of the *j-th* fruit fly to the food sources. The smell concentration value of each fruit fly is calculated by solving the following predefined objective function:

$$Smell_{i}=\mathrm{ObjectiveFunction}(X_{i})$$ (25)

Step 5. Determine the optimal fruit fly: this step determines the best fruit fly by selecting the maximum smell concentration value among the fruit fly swarm.

bestSemllIndex=Max(Smell) (26)

$$X^{best}=X\left(bestSmellIndex\right)$$ (27)

Step 6. Update the fruit fly swarm location: this step will keep the smell concentration value and update the fruit fly swarm location if the current obtained best smell concentration is superior to the previous location.

$$X_{axis}\left(i\right)=X^{best}\left(i\right),i=1,2,\ldots \ldots .,n$$ (28)

Step 7. Check termination conditions and repeat algorithm iterative procedure:

(1) First, compare the current iterative times and the preset maximum iterative times; if the first one reaches the second one, then the termination condition is satisfied and the algorithm’s procedure stops; otherwise, go to the following step.

(2) If the smell concentration value of the current iteration is no longer superior to the smell concentration value of the previous iteration and the current iterative times have reached a predefined value, then the algorithm’s procedure stops; otherwise, go to step 8.

Step 8. Using the proposed mutation strategy to generate a new fruit fly swarm location: from the above step, we have learned that the best smell concentration index with its corresponding location was not changed from the previous generation; to avoid the iterative procedure falling into the trap of a local optimum and to explore a more feasible global best optimal, the proposed mutation strategy has been introduced to address this case. The specific procedure of the mutation strategy is described as follows:

$$\begin{array}{l} t=\left(domain\;of\;dimension\right)\cdot rand()\\ X_{axis}^{t}=minX_{t}+\left(maxX_{t}-minX_{t}\right)\cdot rand() \end{array}$$ (29)

In the above equation, *rand*() is a randomly generated value in the interval [0, 1], and *t* is employed as a variation gene in the range of dimension of the fruit fly. Most intelligent algorithms generally stop the procedure if the current obtained best smell concentration value is not superior to the previous best smell concentration value. However, the proposed mutation strategy continues to have opportunities to find the better solution by changing the fruit fly swarm location.

The pseudo-code of the proposed improved CIFOA is as follows:

**Algorithm 1: CIFOA**

1. **Parameter initialization:**

2.    Initialize *X*_**min**_,*X*_**max**_,*X*,*C*

3. **For** *i* = 0 **to** *d* //*d* is the dimension size

4.    *Xaxis*(*i*) = *minX*(*i*)+(*maxX*(*i*)−*minX*(*i*))∙*rand*( );

5. **EndFor**

6. **For** *j* = 0 **to** *d*

7.    *C*(0,*j*) = **Normlized**(*Xasix*(*j*));

8. **EndFor**

9. **For** *i* = 0 to *n* //*n* is population size

10.    **For** *j* = 0 to *d*

11.        *C*(*i*+1,*j*) = **Logistic**(*C*(*i*,*j*));

12.    **EndFor**

13. **EndFor**

14. **For** *i* = 0 to *n*

15.    **For** *j* = 0 to *d*

16.        *Xaxis*(*i*) = *X*_**min**_(*i*)+(*X*_**max**_(*i*)−*X*_**min**_(*i*))∙*C*(*i*,*j*);

17.    **EndFor**

18. **EndFor**

19. **Calculate fitness value for each fruit fly:**

20. **For** *i* = 0 to *n*

21.    *parameters*_*i*_ = **GetParameter**(*X*(*i*));

22.    *fitness*(*i*) = *CalculateFitness*(*parameters*_*i*_);

23.    *Smell*(*i*) = *fitness*(*i*);

24. **EndFor**

25.    *bestSmellIndex* = **Max**(*Smell*);

26.    *bestX* = *Select*(*X*);

27. **For** *i* = 0 to *n*

28.    *Xaxis*(*i*) = *bestX*(*i*);

29. **EndFor**

30. **Iteration procedure:**

31. **For** *iter* = 0 to *iter*^**max**^

32.    Initialize *C*(0);

33.    Generate a set of *chaos* according to *chaos[0]*;

34.    Update locations according to mutation mechanism;

35.    Calculate fitness value for each fruit fly;

36.    *bestSmellIndex* = **Max**(*Smell*);

37.    **Check termination conditions and perform mutation strategy:**

38.    **IF** *current_iteration* > = *maximum_iteration* **Then**

39.        Stop the procedure;

40.    **EndIF**

41.    **IF** *bestSmell*^*current*^ < *bestSmell*^*previous*^ **Then**

42.        *t* = (*domain of dimension*)∙*rand***();**

43.        *Xaxis*(*t*) = *X*_*min*_(*t*) + (*X*_*max*_(*t*)−*X*_*min*_(*t*))∙*rand***();**

44.    **ElseIF**

45.        **For** *i* = 0 to *d*

46.            *Xaxis*(*i*) = *bestX*(*i*);

47.        **EndFor**

48.    **EndIF**

49. **EndFor**

### 3.3 Testing in several examples

In this subsection, to evaluate and observe the search performance of the proposed CIFOA, this study uses several benchmark functions that are described in Table 1, and FOA, traditional PSO, time-varying particle swarm optimization algorithm (TVPSO), and improved fruit fly optimization algorithm (IFOA) [40] are used as competitors in this testing.

To achieve the available and objective results of the proposed algorithm compared with the other algorithm in this testing, the population size and the maximal iteration number are set to 50 and 1000, respectively. For the parameter setting of traditional PSO, the inertia *W* is set to 1.0 and the acceleration coefficients *C*_1_ and *C*_2_ are set to 2.05 and 2.05, respectively [15, 28]. For the parameter setting of the TVPSO, the lower inertia weight *W*_min_ and the upper inertia weight *W*_max_ are set to 0.5 and 0.9, respectively. The initial *C*_1*i*_,*C*_1*f*_,*C*_2*i*_,*C*_2*f*_ are set to 2.5, 0.5, 0.5 and 2.5, respectively, and *V*_max_ is set to 60% of the upper range of the parameter on each dimension.

In this testing, we implement the proposed CIFOA and other algorithms using C sharp (C#) language with the Visual Studio 2008 platform. To obtain an objective result, this study uses 12 well-known functions in this testing. The dimensions of these optimization functions are set to 30. The specific mathematical description is shown in Table 2, where *lb* and *ub* represent the lower and upper bounds of the solution *X*, respectively.

**Table 2:** The Benchmark functions.

Function ID	Function Name	Equation	Function Typ	Dimension	f(x^*^)	Bounds of *X*
F1	Sphere	$f(x)=\sum_{i=1}^{n}x_{i}^{2}$	Unimodal	30	0	UB(100)
						LB(-100)
F2	Schwefel’s problem 2.22	$f(x)=\sum_{i=1}^{n}\left|x_{i}\right|+\prod_{i=1}^{n}\left|x_{i}\right|$	Unimodal	30	0	UB(10)
						LB(-10)
F3	Quartic	$f(x)=\sum_{i=1}^{n}ix_{i}^{4}+\mathrm{rand}()$	Unimodal	30	0	UB(1.28)
						LB(-1.28)
F4	Sum squares	$f(x)=\sum_{i=1}^{n}ix_{i}^{2}$	Unimodal	30	0	UB(10)
						LB(-10)
F5	Sum of different power	$f(x)=\sum_{i=1}^{n}{\left|x_{i}\right|}^{i+1}$	Unimodal	30	0	UB(1)
						LB(-1)
F6	Rosenbrock	$f(x)=\sum_{i=1}^{n-1}\left(100{\left(x_{i+1}-x_{i}^{2}\right)}^{2}+{\left(x_{i}-1\right)}^{2}\right)$	Unimodal	30	0	UB(30)
						LB(30)
F7	Ackley	$\begin{array}{l} f(x)=-20\exp\left(-0.2\sqrt{\frac{1}{n}\sum_{i=1}^{n}x_{i}^{2}}\right)-\\ \exp\left(\frac{1}{n}\sum_{i=1}^{n}\cos\left(2\pi x_{i}\right)\right)+20+e \end{array}$	Multimodal	30	0	UB(32)
						LB(-32)
F8	Griewank	$f(x)=\frac{1}{4000}\sum_{i=1}^{n-1}x_{i}^{2}-\prod_{i=1}^{n}\cos\left(\frac{x_{i}}{\sqrt{i}}\right)+1$	Multimodal	30	0	UB(600)
						LB(600)
F9	Alpine	$f(x)=\sum_{i=1}^{n}\left\{x_{i}\sin\left(x_{i}\right)+0.1x_{i}\right\}$	Multimodal	30	0	UB(10)
						LB(10)
F10	Powell	$f(x)=\sum_{i=1}^{n/4}\left\{\begin{array}{l} {\left(x_{4i-3}+10x_{4i-2}\right)}^{2}+4{\left(x_{4i-1}+x_{4i}\right)}^{2}+\\ {\left(x_{4i-2}+2x_{4i-1}\right)}^{2}+10{\left(x_{4i-3}+x_{4i}\right)}^{2} \end{array}\right\}$	Multimodal	30	0	UB(5)
						LB(-4)
F11	Rastrigin	$f(x)=\sum_{i=1}^{n}\left(x_{i}^{2}-10\cos\left(2\pi x_{i}\right)+10\right)$	Multimodal	30	0	UB = 5.12
						LB(-5.12)
F12	Solomon problem	$f(x)=1-\cos\left(2\pi \sqrt{\sum_{i=1}^{n}x_{i}^{2}}\right)+0.1\sqrt{\sum_{i=1}^{n}x_{i}^{2}}$	Multimodal	30	0	UB(100)
						LB(-100)

The global optimum of all test functions is equal to F(X^*^) = 0. The lower and upper bounds for functions are set based on their known initial value ranges. For each iteration of the algorithm procedure, the range of values is applied for each parameter *X*_*ij*_. Moreover, we use the closeness criterion to evaluate the error between the searched solution of each algorithm and the final algorithm solution. The error is evaluated using the search space of a well-known function, which is defined as follows:

$$\left|X_{best}-X^{*}\right|\leq \left(upper\_bound-lower\_bound\right)$$ (30)

In the above equation, *X*_*best*_ is the global best solution of the algorithm obtained by each iteration of the algorithm procedure.

In this test, to evaluate the searching performance of five algorithms, the five most frequently used statistical measures, i.e., the best, worst, median, means objective function values, and standard deviation are used to measure the search ability of five algorithms. We use five algorithms to perform 50 independent runs for each optimization function and obtain the statistical results, which are averaged. Moreover, we also present the detailed convergence curves generated by the five algorithms for each optimization function.

Table 3 shows that CIFOA achieves the best results in terms of most performance criteria in comparison with the other intelligent algorithms. In each independent test using the five algorithms, CIFOA almost achieved the optimal solution of each function and obtained significant results in terms of statistical measures. The traditional FOA obtained the worst results in most cases overall, and it could not find the global solution in several function tests. Comparing the CIFOA with the traditional FOA shows that traditional FOA using the proposed chaotic optimization technique in combination with the proposed mutation strategy can enhance the searching ability of both the global and local optimums.

**Table 3:** The testing results of the three algorithms for the benchmark functions.

Benchmark function	algorithm	Best value	Worst value	Median	Means	Std	Average Time
Function1	CIFOA	0	0	0	0	0	0.333530448
	FOA	0.0015	0.00152954	0.0015	0.00150353	8.84E-06	0.52567952
	PSO	46.9277844	81.03940203	68.43008378	66.31215476	9.35121111	0.45106818
	TVPSO	2.57011182	5.2530343	3.78153176	3.78214492	0.59961592	0.44296219
	IFOA	0.00076774	19864.6401877	9616.25445419	9362.67965422	5031.54002686	0.35860204
Function2	CIFOA	1.36E-06	2.24E-06	1.7E-06	1.74E-06	2E-07	0.44376986
	FOA	5.24264069	5.27943571	5.24264069	5.2450921	0.00838239	0.52798553
	PSO	6.65597556	10.60347546	8.3545127	8.46327962	0.82543197	0.48126274
	TVPSO	2.38107103	4.56606483	2.83587925	2.86573324	0.36354308	0.43581129
	IFOA	1.00025496	140.36636921	105.44044879	90.37246037	40.410495	0.38489129
Function3	CIFOA	0.00449358	0.03248204	0.01318236	0.01449095	0.00568992	0.68978405
	FOA	43.30650025	46.71584693	43.30651904	43.49609982	0.66045093	0.59677812
	PSO	0.00321914	0.01473364	0.00747975	0.00815546	0.00279545	0.52370546
	TVPSO	0.01473127	0.08926477	0.04285198	0.04482646	0.01604868	0.5350807
	IFOA	2.40641005	23.99233763	6.74487223	8.03879967	4.12276149	0.41276481
Function4	CIFOA	0	0	0	0	0	0.57697321
	FOA	2.325	2.39218054	2.325	2.33120108	0.01761902	0.5846384
	PSO	7.05952412	23.0391778	10.76372186	11.10410763	2.66095101	0.54813436
	TVPSO	0.44603836	1.45367321	0.74444863	0.79692551	0.22996596	0.42469803
	IFOA	1E-08	4098.6359684	1838.34544005	1659.55963896	1133.78585088	0.42638859
Function5	CIFOA	0	1E-08	1E-08	1E-08	0	0.55225223
	FOA	2.41413989	3394.56423246	2.41413989	70.26851999	474.89938952	0.59365665
	PSO	1.575E-05	0.00015636	6.267E-05	6.729E-05	3.338E-05	0.54133758
	TVPSO	4.2E-06	6.882E-05	2.244E-05	2.244E-05	1.43E-05	0.34650514
	IFOA	4.8E-06	0.51766163	0.08901473	0.14419936	0.16207673	0.42880364
Function6	CIFOA	27.77849226	224.36102113	28.88771013	59.65823219	56.14181148	0.71561607
	FOA	28.93055914	29.20904117	29.18509567	29.17464782	0.03776031	0.65413263
	PSO	535.21563267	2092.59725654	857.02902308	935.18812368	310.56775915	0.61079594
	TVPSO	50.0915007	260.09250574	68.0914448	100.19315468	53.63689523	0.4791295
	IFOA	802.05974855	45938931.0951	727748.6591	5352910.575355	7140960.545249	0.51228287
Function7	CIFOA	6.4E-07	1.08E-06	9E-07	8.9E-07	8E-08	0.57037173
	FOA	0.11422539	0.11543086	0.11422539	0.11429541	0.00025334	0.62419126
	PSO	3.08891225	3.62000336	3.40850015	3.40552595	0.11909537	0.46736982
	TVPSO	0.76043029	1.20173887	1.05696236	1.03287373	0.10292351	0.46470978
	IFOA	1.17177655	19.9050933	15.82120214	15.23704712	3.30422523	0.35988503
Function8	CIFOA	0	0.44987534	0	0.03734983	0.10842514	0.57705417
	FOA	2.78E-06	3.2E-06	2.78E-06	2.81E-06	8E-08	0.4975661
	PSO	1.39171802	1.75384585	1.59891445	1.59947527	0.07868131	0.53438732
	TVPSO	1.01901183	1.04766821	1.03285273	1.03320413	0.00631144	0.36616112
	IFOA	0	236.66796911	81.29755439	79.95490768	45.98983574	0.42762164
Function9	CIFOA	0.01096023	0.02905256	0.01921427	0.01997557	0.00384017	0.63945682
	FOA	0.36200707	0.36648511	0.36200707	0.36251395	0.00127768	0.41726704
	PSO	0.5555723	6.23583619	1.28688615	1.57415173	0.92606614	0.50687815
	TVPSO	0.04691413	1.54387105	0.37571847	0.44654829	0.33720773	0.35788392
	IFOA	2.60785268	36.26263065	14.15463627	14.38035363	6.59076109	0.42133829
Function10	CIFOA	0.03284302	0.37756413	0.12941583	0.13837669	0.06016914	0.6612283
	FOA	16.9428	16.9428	16.9428	16.9428	0	0.4228202
	PSO	11.64524961	48.61811606	22.3435454	24.03177247	7.51416269	0.53200057
	TVPSO	0.42406271	1.10909011	0.81222728	0.83037475	0.15005306	0.38975869
	IFOA	6.78668492	4598.52176616	1397.19974052	1501.61570182	813.57738831	0.44357287
Function11	CIFOA	0	3.98897514	1.01794575	1.68499896	1.11403793	0.5554832
	FOA	104.06037467	107.96149003	106.60893982	106.58501952	0.40733199	0.41106256
	PSO	46.34092901	106.83098785	63.68310374	64.51160886	11.54041507	0.51115152
	TVPSO	24.52376268	167.32843182	57.97808999	61.63611782	27.60762214	0.52063316
	IFOA	47.37883985	266.00413696	122.01030039	140.14453027	49.05889799	0.43695702
Function12	CIFOA	0.09987335	0.59987336	0.29987335	0.28595637	0.1020944	0.40908259
	FOA	0.03333597	0.03396079	0.03333597	0.03336861	0.00013189	0.39189368
	PSO	0.80004655	1.09991055	0.92589667	0.94091837	0.05730999	0.42242791
	TVPSO	0.29987827	0.40146745	0.39987345	0.35680167	0.04630805	0.41691559
	IFOA	7.63407942	23.40037258	12.72564107	13.46440787	3.27607954	0.32941129

Moreover, CIFOA showed good performance in several complex multimodal functions, while the other four algorithms achieved worse results in terms of the median and standard deviation. In addition, CIFOA was also shown to be an efficient and robust algorithm for solving continuous functions.

**Fig 2:**
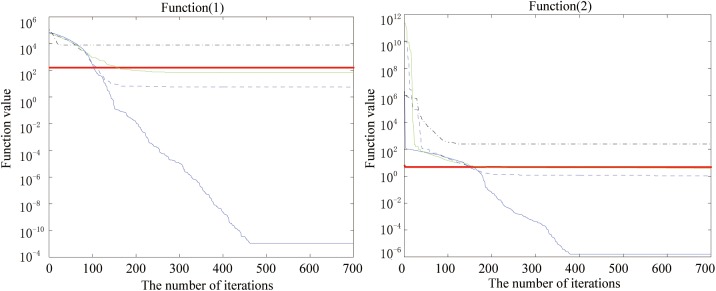
The fitness value of various algorithms for Sphere and Schwefel’s problem 2.22 during increasing iterative times.

**Fig 3:**
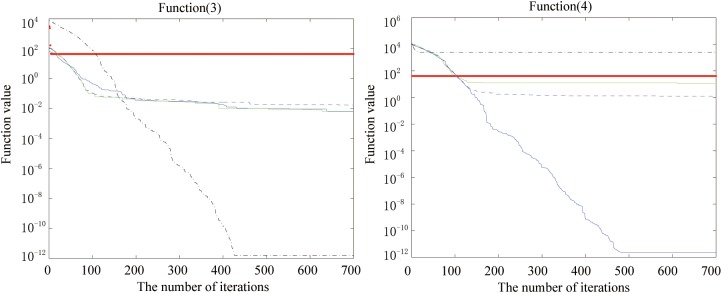
The fitness value of various algorithms for Quartic and Sum squares during increasing iterative times.

**Fig 4:**
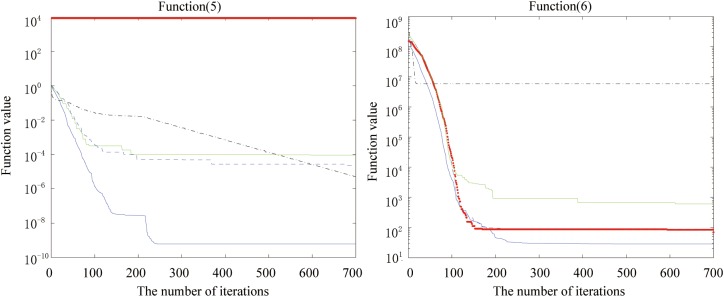
The fitness value of various algorithms for Rosenbrock and Sum of different power during increasing iterative times.

**Fig 5:**
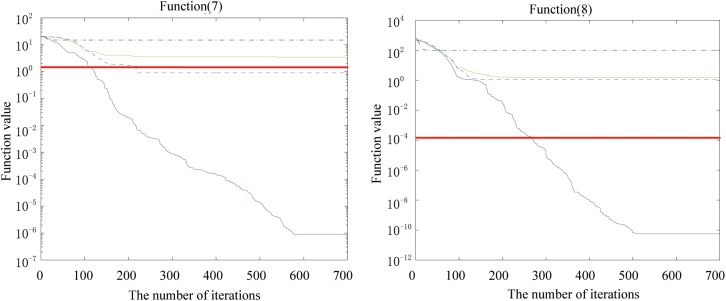
The fitness value of various algorithms for Ackley and Griewank during increasing iterative times.

**Fig 6:**
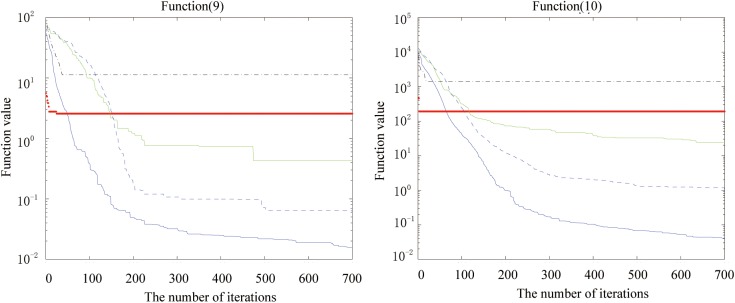
The fitness value of various algorithms for Alpine and Powell during increasing iterative times.

**Fig 7:**
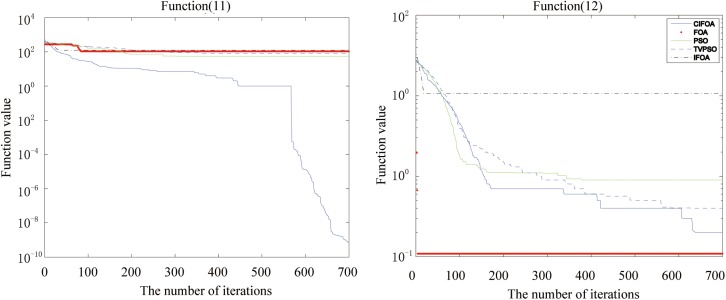
The fitness value of various algorithms for Rastrigin and the Solomon problem during increasing iterative times.

Figs 2, 3, 4, 5, 6 and 7 shows the convergence curves generated by the five intelligent algorithms for solving different complex nonlinear continuous functions. To observe the convergence curves generated by five algorithms, we have found that the proposed algorithm always successfully reached the closest solution of the optimization function with a minimal number of iterations. In particular, when solving the more complicated mathematical equations, the proposed algorithm has better searching performance than the other intelligent algorithms. Thus, it can also be concluded that the mutation strategy and chaotic PSO can help the algorithm simultaneously search for the global and local optimums, whereas the mutation strategy also allows the algorithm procedure to jump out of the local extremum.

Furthermore, this testing compares the proposed algorithm CIFOA with other well-known algorithms. The testing results show that the proposed CIFOA is significantly better than other algorithms presented for solving the complex nonlinear continuous functions.

## 4. Experiments and applications

In this section, we applied the proposed CIFOA to optimize the SVM, aiming to enhance the classification performance of the SVM classifier for solving real-world classification problems. In this proposed method, namely, CIFOA-SVM, which simultaneously performs SVM model parameter setting turning (penalty parameter C and hyperplane parameters) and feature selection, attempts to achieve an optimal SVM model, which has better generalization ability and excellent effectiveness for real-world classification tasks. To evaluate the classification performance of the proposed methods, this study has constructed comparative experiments that were performed between the proposed CIFOA-SVM and other intelligent methods, including GA-SVM, PSO-SVM, FOA-SVM, and TVPSO-SVM. Moreover, two real-world problems have been introduced in this study: the medical diagnosis problem and the credit card problem.

### 4.1 Fitness function design

A well-designed fitness function can explore a more optimal global best solution and avoid falling into the trap of local optima. Although different performance criteria have been proposed to evaluate the classification performance of a SVM classifier, the most popular and frequently used of these performance criteria are sensitivity and specificity. To explain the effect of sensitivity and specificity in the performance metrics, we introduce the confusion matrix that is displayed in Table 4.

**Table 4:** Confusion matrix.

Classification	classified	
	Testing Result Positive	Testing Result Negative
Actual Positive Samples	True Positive (TP)	False Negative (FN)
Actual Negative Samples	False Positive (FP)	True Negative (TN)

From the above the confusion matrix, true positive (TP) means samples correctly labeled as a positive class and true negative (TN) means samples correctly labeled as a negative class. False positive (FP) means that samples are incorrectly classified as a negative class and false negative (FN) means that the negative samples are incorrectly classified as a positive class. A well-performing classifier should have a high true positive (TP) and a low false positive (FP). Moreover, the number of selected features and support vectors also play an important role in the classification performance of the SVM classifier. The main reasons are clear that a small feature space can reduce the complexity of the procedure of training and prediction, and a small number of support vectors can avoid over-fitting and improve the classification accuracy for the SVM classifier. Thus, the four performance criteria are understood to design a weighted objective function that simultaneously considers the trade-off between sensitivity and specificity, maximizing the true positive rate and minimizing (1 –the false positive rate), along with the number of selected features and support vectors. The detailed proposed fitness function is depicted as follows:

$$\begin{array}{l} \mathrm{FitnessFunction}(X)=W_{sen}\cdot \left(\frac{TP}{TP+FN}\right)+W_{1-spe}\cdot \left(1-\frac{FP}{FP+TN}\right)\\ +W_{F}\cdot \left(1-\frac{\sum_{i=1}^{N_{f}}F_{i}}{N_{f}}\right)+W_{s}\cdot \left(1-\frac{\sum_{i=1}^{S_{f}}S_{i}}{S_{f}}\right) \end{array}$$ (31)

Considering that any of these four components of the fitness function have different effects on the classification performance of the SVM classifier, we designed the fitness function using multiple performance criteria to convert a single weighted criterion, i.e., the sensitivity, specificity, and the number of selected features and support vectors are converted into one by combining their weight values *W*_*sen*_,*W*_1−*spe*_,*W*_*F*_,*W*_*S*_. In the above equation, *F*_*i*_ represents the value of the i-th feature mask, with a value of “1” meaning that it is selected as one part of the input feature space; otherwise, it is ignored during the training phase. *N*_*f*_ refers to the number of total features.

### 4.2 Data representation

To implement the proposed CIFOA-SVM, the radial basis function (RBF) is employed as the kernel function of the SVM classifier because it can effectively address high-dimensional data, and only one parameter is required to be optimized. The selected feature subset and model parameter setting are generally represented by a binary string or other representation. In the GA, one of the most frequently used of these representations is the binary string, which can easily map the selection state of features to a feature mask. By contrast, in FOA, the sigmoid function and random strategy are used to convert the distance *X* into a feature mask (“1” means that the feature is selected as the input feature, and “0” means that the feature is ignored). The specific equation is described as follows:

$$\begin{array}{l} \mathrm{SigFun}(X_{ij})=\frac{1}{1+e^{\left(-X_{ij}\right)}},j=1,2,\ldots ,m\\ F_{ij}=\left\{ \begin{array}{l} 1,\;\text{if}\;\mathrm{rand}()<\mathrm{SigFun}(X_{ij})\\ 0,\;\text{if}\;\text{rand}()\geq \mathrm{SigFun}(X_{ij}) \end{array}\right.,j=1,2,..,m \end{array}$$ (32)

where rand() is a randomly generated value in the range [0, 1].

### 4.3 The proposed CIFOA-SVM framework

**Fig 8:**
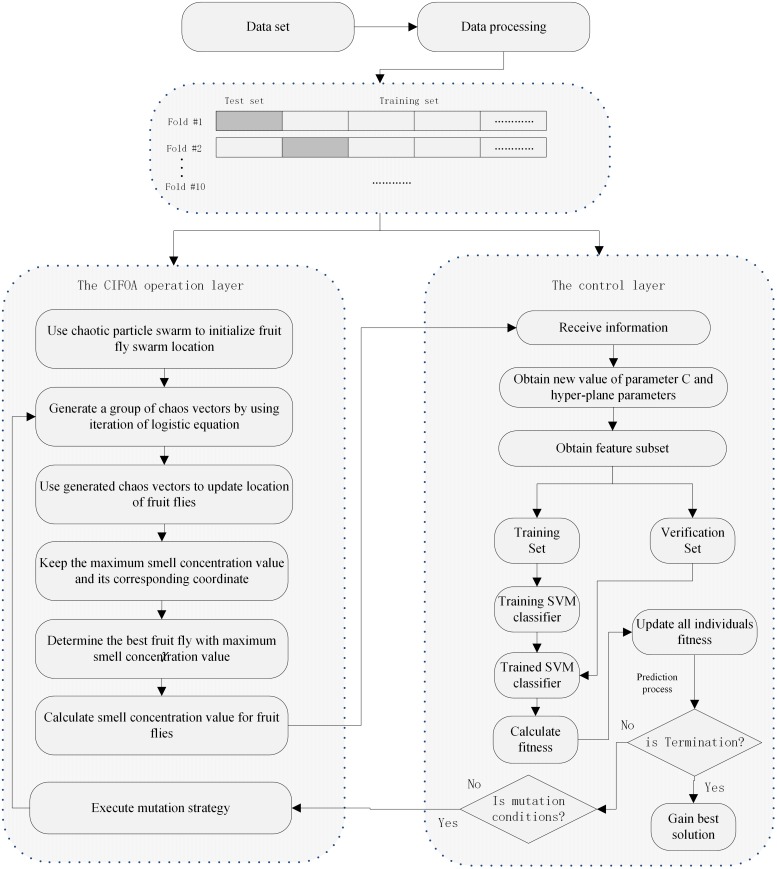
The basic process of the proposed framework.

This section describes the detailed procedure of the proposed CIFOA-SVM framework. Based on the nature of the proposed framework, its procedure mainly consists of two stages. One of these stages is the algorithm computing processing layer, which is used to implement CIFOA operations, such as population initialization, fruit fly swarm location update, and maintaining the maximum smell concentration and its corresponding location. Another of these stages is the microcosmic computing layer, which is also called the control layer in this paper. This stage is mainly responsible for the calculation of the smell concentration value of each individual using SVM and the proposed evaluation function. The detailed basic procedure of the proposed framework is shown in Fig 8.

To give a more detailed description of how an optimal SVM model is achieved, we provide a pseudo-code of the proposed CIFOA-SVM framework as follows:

**Algorithm 2: CIFOA-SVM**

1. **Parameter initialization:**

2.    Initialize *X*_**min**_,*X*_**max**_,*X*,*C*

3. **For** *i* = 0 **to** *d* //*d* is the dimension size

4.    *Xaxis*(*i*) = *minX*(*i*)+(*maxX*(*i*)−*minX*(*i*))∙*rand*( );

5. **EndFor**

6. **For** *j* = 0 **to** *d*

7.    *C*(0,*j*) = **Normalized**(*Xasix*(*j*));

8. **EndFor**

9. **For** *i* = 0 to *n* //*n* is population size

10.    **For** *j* = 0 to *d*

11.        *C*(*i*+1,*j*) = **Logistic**(*C*(*i*,*j*));

12.    **EndFor**

13. **EndFor**

14. **For** *i* = 0 to *n*

15.    **For** *j* = 0 to *d*

16.        *Xaxis*(*i*) = *X*_**min**_(*i*)+(*X*_**max**_(*i*)−*X*_**min**_(*i*))∙*C*(*i*,*j*);

17.    **EndFor**

18. **EndFor**

19. **Calculate fitness value for each fruit fly:**

20. **For** *i* = 0 to *n*

21.    *model_parameters* = **GetParameter**(*X*(*i*));

22.    *feature_mask* = **GetFeatures**(*X*(*i*));

23.    Train SVM by using model parameters and selected features;

24.    Make a prediction by using this model;

25.    *Smell*(*i*) = **FitnessFunction**(*features*,*results*);

26. **EndFor**

27.    *bestSmellIndex* = **Max**(*Smell*);

28.    *bestX* = *Select*(*X*);

29. **For** *i* = 0 to *n*

30.    *Xaxis*(*i*) = *bestX*(*i*);

31. **EndFor**

32. **Iteration procedure:**

33. **For** *iter* = 0 to *iter*^**max**^

34.    Initialize *C*(0);

35.    Generate a set of *chaos* according to *C*(0);

36.    Update locations according to mutation mechanism;

37.    Calculate fitness value for each fruit fly;

38.    *bestSmellIndex* = **Max**(*Smell*);

39.    **Check termination conditions and perform mutation strategy:**

40.    **IF** *current_iteration* > = *maximum_iteration* **Then**

41.        Stop the procedure;

42.    **EndIF**

43.    **IF** *bestSmell*^*current*^ > = *bestSmell*^*previous*^ **Then**

44.        **For** *i* = 0 to *d*

45.            *Xaxis*(*i*) = *bestX*(*i*);

46.        **EndFor**

47.    **ElseIF**

48.        *t* = (*domain of dimension*)∙*rand***();**

49.        *Xaxis*(*t*) = *X*_*min*_(*t*)+(*X*_*max*_(*t*)−*X*_*min*_(*t*))∙*rand***();**

50.    **EndIf**

51. **EndFor**

### 4.4 Parameter setting and datasets

In this section, we describe the parameter setting of various methods and the property of datasets in detail. To evaluate the classification performance of the proposed framework compared with that of the other methods in the classification tasks, GA, FOASVM, PSOFS, and TVPSOFS have been introduced to be used as the competitors in these experiments. GAFS means that the genetic algorithm (GA) is used to simultaneously turn the model parameter setting and select the feature subset. FOASVM means that the FOA is used to turn the parameter setting of the SVM classifier without selecting the feature subset. PSOFS means that the traditional PSO algorithm is used to adjust the model parameter setting and to select the feature subset simultaneously. TVPSOFS means that the traditional PSO uses a time-varying technique to dynamically adjust its inertia weight and coefficients based on iterative times. To achieve an objective comparison in various methods, the same population size and maximum iteration number are applied for all methods. For the parameter setting of the GAFS, the crossover probability rate and mutation probability rate are set to 0.75 and 0.15, respectively. The binary string is used to represent the individual, and the penalty parameter C and hyper-parameters are represented by two binary strings, with each binary string being composed of 20 bit (2^20^), which means that the searching precision of two parameters depends exclusively on the length of the binary string. The roulette wheel selection and elite selection strategy are used to generate new individuals that merge with the best individual into the new population of the next generation. For the parameter setting of FOA, the lower and upper bounds of the flight range are set to -10 and 10, respectively. The location of each fruit fly is limited to the range of [–10, 10]. For the parameter setting of the traditional PSO algorithm, the acceleration coefficients *C*_1_,*C*_2_ are set to 2.05 and 2.05 [15, 18]. The inertia weight *W* is set to 0.729 [59]. The lower and upper bounds of particle velocity are set to 0 and 1, respectively, for the feature mask, and set to 60% of the range of the parameter on each dimension for model parameters. For the parameter setting of the time-varying particle swarm optimization algorithm (TVPSO), the initial acceleration coefficients *C*_1*i*_,*C*_1*f*_,*C*_2*i*_,*C*_2*f*_ are set to 2.5, 0.5, 0.5, and 2.5, respectively. The initial inertia weights *W*_min_,*W*_max_ are set to 0.4 and 0.9, respectively. The lower and upper bounds of penalty parameter *C* are set to 0 and 1000, respectively. The lower and upper bounds of the hyperplane parameter are set to 0 and 10, respectively.

To achieve a feasible result and avoid overtraining, we introduce the k-fold crossover-validation technique in this experiment. The k-fold crossover-validation technique is the most popular and frequently used method in myriad classification experiments. The main idea behind this technique is to divide the original training dataset into several subsets; each subset is completely independent and is constituted by the same number of samples. In each run of the k-fold crossover-validation, each subset has the same chance to be used as the testing dataset, and the remaining k-1 subsets are used as the training dataset. In this study, we set k = 10 for k-fold crossover-validation, i.e., we divide the original training dataset into ten parts; and each part can be used as the testing dataset and the remaining 9 parts are used as the training dataset. Finally, the training subset, SVM model parameters, and selected feature subset are fed into the SVM to generate an SVM model; then, the testing subset is used with the SVM model to make a prediction, and the fitness value is calculated based on the obtained classification accuracy and other performance criteria. This study uses five methods to perform ten iterations of 10-fold crossover-validation on the various datasets, and the obtained results are averaged. To generate enough classification performance for comparison, this study repeatedly performs 10 iterations for the 10-fold crossover-validation procedure instead of a single repetition of the 10-fold crossover-validation procedure.

### 4.5 Datasets and data preprocessing

Many datasets contain several dissimilar properties. To avoid the feature value spanning over great numerical ranges and dominating the other features in smaller numerical ranges–and to address numerical other difficulties in the calculation–the data normalized method has been introduced to scale all the input variables of the datasets into the range [0, 1] or [–1, 1]. The main advantage of scaling variables is that it can help improve the classification performance of the SVM classifier and reduce the running time of training and prediction. The specific data normalized method is described as follows:

$$V_{ij}^{'}=\frac{V_{ij}-\min(V_{i})}{\max(V_{i})-\min(V_{i})},j=1,2\ldots ..,d.$$ (33)

In the above equation, *V*_*ij*_ is the feature value of the j-th item of the *i-th* row recorded. The function min() is used to find the smallest feature value among all the features of the *i-th* row recorded, and the function max() is used to select the greatest feature value among all the features of the *i-th* row recorded. $V_{ij}^{'}$ is the transformed feature whose value is restricted in the [0, 1] range.

### 4.6 The medical diagnosis problem

To investigate the classification performance of various methods in terms of solving the medical diagnosis problem, three datasets have been employed as the experimental dataset, all from the UCI machine learning database repository. These datasets include the Wisconsin Diagnostic Breast Cancer (WDBC-1995), Pima Indians Diabetes (PID), and the Parkinson’s disease dataset (PDD). The WDBC-1995 contains 569 samples (212 malignant and 357 benign samples) represented by 30 continuous features [60]. This breast cancer database was established by Dr. William H. Wolberg from the University of Wisconsin Hospitals and donated in November 1995. Ten real-valued features are computed for each cell nucleus. The details of the 32 attributes of the (WBC-1995) dataset are presented in Table 5. The PID [61] consists of 758 samples (500 normal samples and 268 diabetes samples), represented by 8 attributes. The PID was constructed by collecting donative diabetes cases in which all patients were females at least 12 years old of Pima Indian heritage. The detailed attributes and categories of this dataset are shown in Table 6. The PDD [62] was created and collected by Max Little of the University of Oxford, in collaboration with the National Centre for Voice and Speech, Denver. This dataset contains 195 samples (147 non-disease samples and 48 disease samples) represented by 23 attributes. These attributes are various biomedical voice measurements for 23 patients with Parkinson’s disease and 8 healthy individuals. The primary purpose of this dataset is to discriminate healthy people from those with Parkinson’s disease. The detailed 23 attributes of the dataset are shown in Table 7.

**Table 5:** Wisconsin Diagnostic Breast Cancer (WDBC-1995).

Feature ID	Feature Name	Domain
1	Sample code number	ID_number
2	Diagnosis	1–32
3–32	Cell information	1–32

**Table 6:** Pima Indians Diabetes (PID).

Feature ID	Feature Name	Domain
1	Number of times pregnant	1–8
2	Plasma glucose concentration at 2 hours in an oral glucose tolerance test	1–8
3	Diastolic blood pressure (mm Hg)	1–8
4	Triceps skin fold thickness (mm)	1–8
5	2-hour serum insulin (mu U/ml)	1–8
6	Body mass index (weight in kg/(height in m)^2)	1–8
7	Diabetes pedigree function	1–8
8	Age	1–8
9	Class variable	

**Table 7:** The attributes of Parkinson’s disease dataset (PDD).

Feature ID	Feature Name	Description
1	Name	ASCII subject name and recording number
2	MDVP:Fo(Hz)	Average vocal fundamental frequency
3	MDVP:Fhi(Hz)	Maximum vocal fundamental frequency
4	MDVP:Flo(Hz)	Minimum vocal fundamental frequency
5	MDVP:Jitter(%)	Several measures of variation in fundamental frequency
6	MDVP:Jitter(Abs)	
7	MDVP:RAP	
8	MDVP:PPQ	
9	Jitter:DDP	
10	MDVP:Shimmer	Several measures of variation in amplitude
11	MDVP:Shimmer(dB)	
12	Shimmer:APQ3	
14	MDVP:APQ	
15	Shimmer:DDA	
16	NHR	
17	HNR	
18	status	
19	RPDE	Two measures of ratio of noise to tonal components in the voice
20	DFA	Signal fractal scaling exponent
21	spread1	Three nonlinear measures of fundamental frequency variation
22	spread2	
23	D2	
24	PPE	

There are several performance metrics to evaluate the classification performance of the proposed approach, including the classification accuracy, sensitivity, specificity, the number of selected features and support vectors, and the running time of the training and prediction procedure that have been employed to construct a comprehensive comparative experiment. In each run of various datasets of this experiment, we undertake the five methods and perform 10 iterations of 10-fold crossover-validations, and the obtained experimental results are averaged.

The detailed classification results of the five methods in terms of the average classification accuracy, sensitivity, specificity, and number of selected features and support vectors on the Wisconsin Diagnostic Breast Cancer (WDBC-1995) database are presented in Table 8. We first compare the classification accuracy, sensitivity, and specificity. As shown in this table, the proposed CIFOA-SVM has achieved average results of 98.21% classification accuracy, 99.51% sensitivity, and 96.11% specificity. These obtained statistic results show that the proposed CIFOA-SVM outperforms other methods in terms of classification accuracy and in the trade-off between sensitivity and specificity. Moreover, this table also shows that not only did the proposed CIFOA-SVM achieve the best results in terms of classification accuracy but also the standard deviation produced by the CIFOA-SVM was also much smaller in terms of classification accuracy, sensitivity and specificity than that of other methods. This result indicates that the proposed CIFOA-SVM can obtain considerably more consistent and smooth prediction results than other methods for different fold runs of 10-fold crossover-validation. To compare the number of selected features of the optimal SVM model using various outcomes, the proposed CIFOA-SVM almost requires the least features to construct an optimal SVM model among the FOA-SVM, PSOFS, and TVPSO. Table 8 also shows that feature methods have better classification accuracy than FOASVM, which indicates that feature selection actively improves the accuracy of the SVM classifier for solving medical diagnosis problems. For the number of support vectors of the optimal SVM model constructed using a variety of methods, the best SVM model with the fewest support vectors was achieved by the proposed CIFOA-SVM. Moreover, the same results are shown in Tables 9 and 10.

**Table 8:** Classification results of various methods in terms of classification accuracy, sensitivity, specificity, number of selected features and support vectors, and model parameters for the WBC-1995.

Criteria	Methods				
	GAFS	PSOFS	TVPSOFS	FOASVM	CIFOA-SVM
Sensitivity	0.9948 + 0.0001	0.9889 + 0.0003	0.9903 + 0.0002	0.9666 + 0.0007	0.9951 + 0.0002
Accuracy	0.9803 + 0.0004	0.9553 + 0.0004	0.9642 + 0.0007	0.9196 + 0.0006	0.9821 + 0.0001
Specificity	0.9662 + 0.0013	0.8964 + 0.0039	0.9326 + 0.0020	0.8366 + 0.0045	0.9611 + 0.0014
N of features	11.5 + 4.05	17.2 + 10.25	15.9 + 9.09	30 + 0.0	15.4 + 1.24
N of SVs	188.8	189.4	189.7	199.8	157.6
Parameter *C*	464.4346	503.2890	516.4186	418.5625	392.5621
Parameter *γ*	0.3340	0.6081	0.0100	0.0399	0.0548

**Table 9:** Classification results of various methods in terms of classification accuracy, sensitivity, specificity, number of selected features and support vectors, and model parameters for Pima Indians Diabetes (PID).

Criteria	Methods				
	GAFS	PSOFS	TVPSOFS	FOASVM	CIFOA-SVM
Sensitivity	0.4658 + 0.0026	0.8739 + 0.0011	0.9208 + 0.0024	0.8921 + 0.0029	0.9174 + 0.0023
Accuracy	0.7473 + 0.0026	0.7474 + 0.0010	0.7552 + 0.0011	0.7460 + 0.0017	0.7592 + 0.0005
Specificity	0.9001 + 0.0039	0.5012 + 0.0316	0.4434 + 0.0207	0.4733 + 0.0095	0.4689 + 0.0097
N of features	5.3 + 1.41	5.8 + 1.36	4.3 + 1.41	8.0 + 0.0	5.7 + 0.81
N of SVs	450.2	410.1	490.1	524.5	489.1
Parameter *C*	337.58	482.10	300.20	741.60	236.35
Parameter *γ*	0.3340	0.5708	0.5253	0.0232	0.4517

**Table 10:** Classification results of various methods in terms of classification accuracy, sensitivity, specificity, number of selected features and support vectors, and model parameters for Parkinson’s disease dataset (PDD).

Criteria	Methods				
	GAFS	PSOFS	TVPSOFS	FOASVM	CIFOA-SVM
Sensitivity	0.9890 + 0.0017	0.9703 + 0.0019	0.6533 + 0.0016	0.9790 + 0.0029	0.9810 + 0.0013
Accuracy	0.9621 + 0.0022	0.9157 + 0.0024	0.9473 + 0.0011	0.9615 + 0.0017	0.9631 + 0.0006
Specificity	0.9352 + 0.0069	0.8333 + 0.0187	0.8666 + 0.0433	0.9440 + 0.0025	0.9452 + 0.0027
N of features	10.2 + 6.92	15.3 + 3.61	12.5 + 8.45	22.0 + 0.0	12.1 + 1.59
N of SVs	78.1	82.6	81.2	129.8	75.4
Parameter *C*	594.1864	449.5192	562.3227	543.8755	472.205
Parameter *γ*	0.4939	0.5348	0.3189	0.3495	0.4518

**Fig 9:**
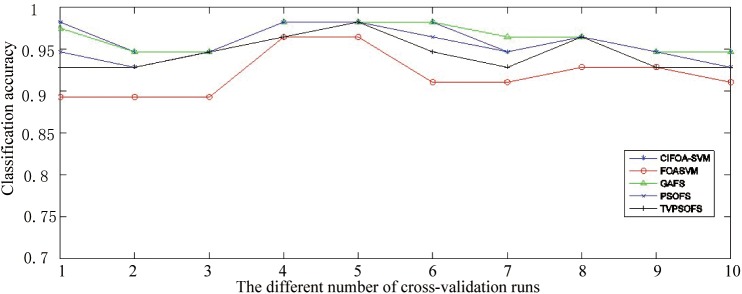
The classification accuracy achieved for each fold run by various methods for the WDBC-1995.

Fig 9 displays the classification accuracy obtained by various methods for each fold run of 10-fold crossover-validation on the WDBC-1995 datasets. This figure shows that in most of the fold runs of 10-fold crossover-validation on the WDBC-1995 dataset, CIFOA-SVM obtained significantly better results than other methods in terms of classification accuracy.

**Fig 10:**
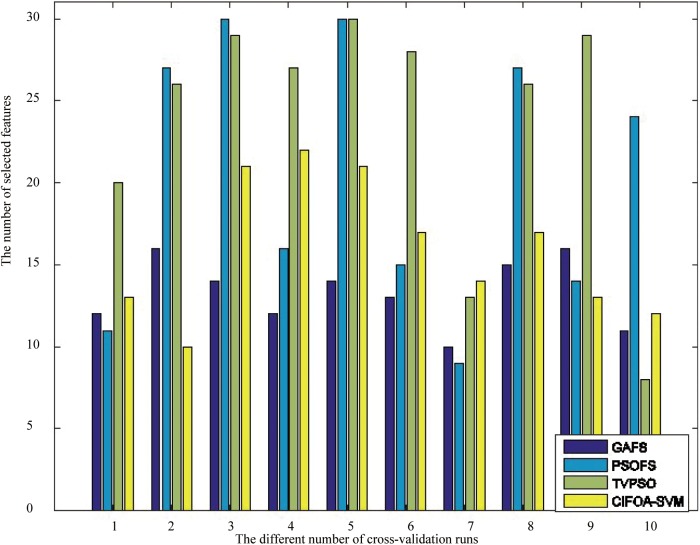
The results of the five methods in terms of the number of selected features for the WDBC-1995.

To investigate the effects of the number of selected features on the classification accuracy of the proposed framework compared with other methods in solving medical diagnosis problems, we perform one iteration of 10-fold cross-validation using the five methods on the WDBC-1995 dataset. The detailed results are displayed in Fig 10. This graph shows that the feature selection methods, including GAFS, CIFOA-SVM, PSOFS and TVPSOFS, can improve classification accuracy better than the ordinary parameter tuning FOASVM in different fold runs of 10-fold crossover-validation. The main reason for these results is that some medical data contain redundancies and useless features, including noise or irrelevant feature information, which might affect the quality and efficiency of adjusting parameters for the SVM classifier. Thus, simultaneous feature selection and parameter optimization can enhance the classification accuracy for classifying medical data. Furthermore, CIFOA-SVM almost requires fewer selected features to construct an optimal SVM model than the PSOFS and TVPSO at each fold run of 10-fold cross-validation for the (WBC-1995) dataset, which indicates that CIFOA-SVM filters out most of the irrelevant features from the SVM classifier, reducing its complexity and improving its classification performance.

**Fig 11:**
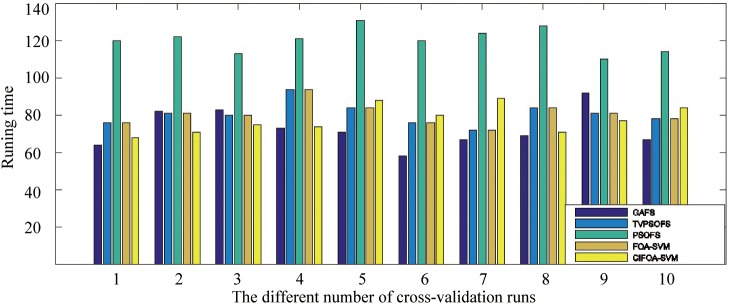
The running time required for each run by the five methods for the WDBC-1995.

To investigate the computational time of the five methods required to implement the training and prediction procedure, we recorded the five methods’ running time for each fold run of 10-fold crossover-validation using the WDBC-1995 database. Fig 11 displays the computational time in seconds under the five methods to perform a one-fold run of the 10-fold crossover-validation. This figure shows that the proposed CIFOA-SVM requires an average of almost 150 s to implement the training of an optimal SVM model and to make a prediction in each fold for WDBC-1995. Compared with the proposed CIFOA-SVM and GAFS, the computational time was reduced by 4 seconds using CIFOA-SVM. Compared with the proposed CIFOA-SVM and PSOFS, the computational time in seconds was reduced by 43 seconds by CIFOA-SVM. Compared with the proposed CIFOA-SVM and TVPSO, the computational time was reduced by 9 seconds using CIFOA-SVM. Compared with the proposed FOASVM and the four other methods based on the feature selection technique, FOASVM achieves an optimal SVM model with the required minimal computational resources. Moreover, traditional PSO requires the most running time to implement an optimal SVM model. The above results show that our proposed CIFOA-SVM algorithm is an efficient framework among the feature selection methods for the medical data classification tasks.

**Fig 12:**
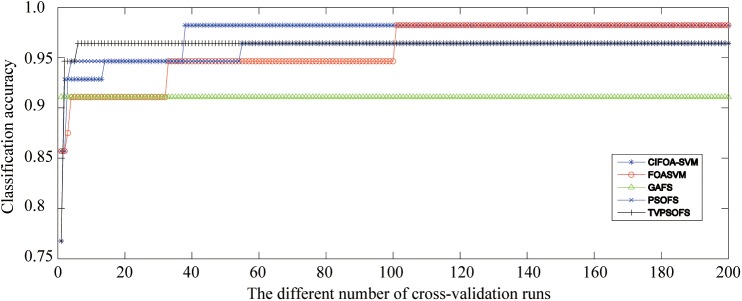
The convergence curves generated for the fold #3 run by the five methods for the WDBC-1995.

Fig 12 presents the convergence curves generated by the five methods for different numbers of iterations for the fold #3 run of the 10-fold crossover-validation using the WDBC-1995 database. These curves show that not only did the proposed CIFOA-SVM obtain better classification accuracy during the different numbers of iterations but also the convergence procedure of CIFOA-SVM determined the final optimal SVM model and reached the stopping criteria much more quickly than PSOFS, TVPSO, FOASVM, and GAFS. This result indicates that the proposed CIFOA-SVM has excellent searching ability for determining an optimal feature subset and a proper parameter setting for the SVM classifier.

### 4.7 Credit card problem

To evaluate the classification performance of the five methods for solving the credit card problem, we selected two datasets, both of which come from the UCI machine learning database repository. One of these datasets is the German Credit Data (GCD) [63], which contains 1000 records (300 bad credit risk records and 700 good credit records) represented by 20 numeric and non-numeric features. The information regarding these features is illustrated in Table 11. To convert the original non-numeric features to numeric features that SVM recognizes, we adopt a real number to represent non-numeric values of the discrete feature. The GCD is used to evaluate credit card risks, which are either good or bad credit risks. The other dataset is the Australian Credit Approval (ACA) [64], which consists of 690 samples (307 positive samples and 383 negative samples) represented by 14 attributes. The main purpose of ACA is to consider credit card applications.

**Table 11:** The detailed attributes of German Credit Data (GCD).

Feature ID	Feature Name	Data Type
1	Qualitative	non-numeric
2	Duration in months	numerical
3	Credit history	non-numeric
4	Purpose	non-numeric
5	Credit amount	Numerical
6	Savings account/bonds	non-numeric
7	Diabetes pedigree function	non-numeric
8	Installment rate in percentage of disposable income	non-numeric
9	Personal status and sex	non-numeric
10	Other debtors / guarantors	non-numeric
11	Present residence since	numerical
12	Property	non-numeric
13	Age in years	numerical
14	Other installment plans	non-numeric
15	Housing	non-numeric
16	Number of existing credits at this bank	numerical
17	Job	non-numeric
18	Number of people being liable to provide maintenance for	non-numeric
19	Telephone	non-numeric
20	Foreign worker	non-numeric

**Fig 13:**
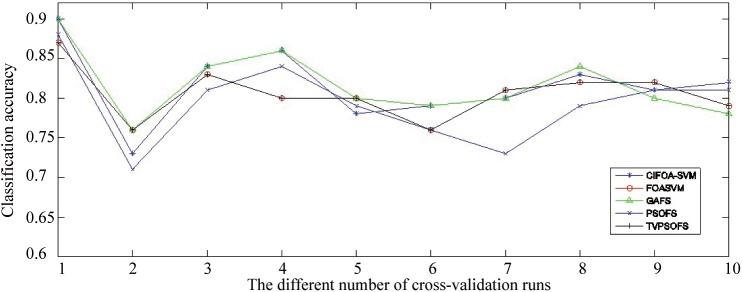
The classification accuracy achieved for each fold run by various methods for German Credit Data (GCD).

To evaluate the classification performance of the proposed method in solving the credit card problem, experiments using the five methods for each fold run of the 10-fold crossover-validation on the GCD and ACA datasets have been performed. All the results obtained, including classification accuracy, sensitivity, specificity, and number of selected features and support vectors, were averaged. Tables 12 and 13 display the detailed classification results achieved by the five methods for 10 runs of 10-fold crossover-validation. Table 13 shows that the proposed CIFOA-SVM has achieved average results of 81.70% classification accuracy, 95.03% sensitivity, 51.04% specificity, 16.1 selected features, and 504.3 support vectors. To compare the CIFOA-SVM with other methods in terms of the GCD, not only did the CIFOA-SVM obtain the best results with respect to most of the performance criteria but also the standard deviation generated by CIFOA-SVM is much smaller in terms of classification accuracy and the number of selected features than that of other methods. Table 12 presents the same phenomenon. Moreover, Fig 13 shows the classification accuracy achieved by the five methods for each fold run of the 10-fold crossover-validation on the GCD. This graph shows that the proposed CIFOA-SVM almost achieves better results than the other methods in terms of classification accuracy in each fold for the GCD datasets. The results achieved above by the five methods for the two datasets indicate that the proposed method has better classification performance in solving credit card problems.

**Table 12:** Classification results of various methods in terms of classification accuracy, sensitivity, specificity, number of selected features and support vectors, and model parameters for German Credit Data (GCD).

Criteria	Methods				
	GAFS	PSOFS	TVPSOFS	FOASVM	CIFOA-SVM
Sensitivity	0.9213 + 0.0026	0.9246 + 0.0006	0.9359 + 0.0008	0.9282 + 0.0006	0.9503 + 0.0007
Accuracy	0.8150 + 0.0014	0.7980 + 0.0013	0.8060 + 0.0016	0.8160 + 0.0019	0.8170 + 0.0006
Specificity	0.5524 + 0.0009	0.5027 + 0.0105	0.5059 + 0.0018	0.5548 + 0.0015	0.5104 + 0.0008
N of features	17.1 + 6.49	15.3 + 28.1	16.4 + 23.4	24.0 + 0.0	16.1 + 3.09
N of SVs	540.965	557.9	527.6	518.1	504.3
Parameter *C*	161.4932	166.4563	146.5901	747.4506	166.3319
Parameter *γ*	0.3754	0.6177	0.5136	0.0287	0.6666

**Table 13:** Classification results of various methods in terms of classification accuracy, sensitivity, specificity, number of selected features and support vectors, and model parameters for Australian Credit Approval (ACA).

Criteria	Methods				
	GAFS	PSOFS	TVPSOFS	FOASVM	CIFOA-SVM
Sensitivity	0.9429 + 0.0003	0.8289 + 0.0020	0.8152 + 0.0050	0.7552 + 0.0177	0.8376 + 0.0015
Accuracy	0.8200 + 0.0015	0.7991 + 0.0101	0.8231 + 0.0017	0.8115 + 0.0032	0.8376 + 0.0007
Specificity	0.5382 + 0.0093	0.8513 + 0.0057	0.8315 + 0.0042	0.8545 + 0.0052	0.8190 + 0.0018
N of features	8.1 + 3.69	8.3 + 6.01	6.5 + 2.35	14.0 + 0.0	6.3 + 1.29
N of SVs	540.8	402.2	399.5	384.8	403.2
Parameter *C*	105.1754	480.7929	439.4262	418.5625	401.1397
Parameter *γ*	0.5761	0.4315	0.4163	0.0399	0.5574

**Fig 14:**
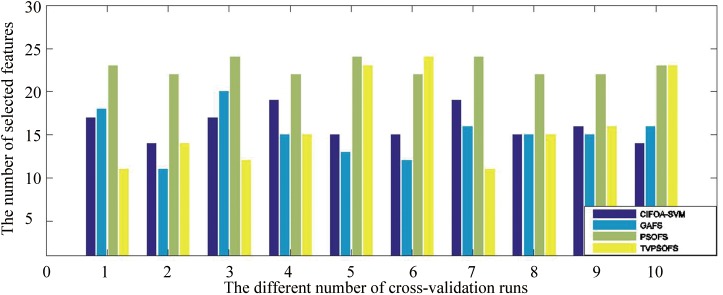
The results of the five methods in terms of the number of selected features for German Credit Data (GCD).

Feature selection is one of the important effect factors for the classification accuracy of the SVM classifier. According to Table 12 and Fig 14, feature selection methods have better classification accuracy than the parameter adjustment methods, although FOA has better searching ability for the optimal SVM parameters among these parameter adjustment methods. Fig 14 shows the number of selected features of the five methods for 10 runs of 10-fold crossover-validation for the GCD. As shown, the proposed CIFOA-SVM almost requires the least number of selected features to implement an optimal SVM model compared with the PSOFS and TVPSO at each fold run of 10-fold cross-validation for the GCD dataset.

**Fig 15:**
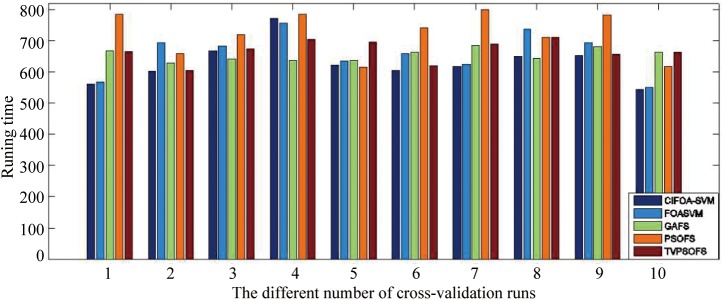
The required running time in seconds for each run by the five methods for German Credit Data (GCD).

To investigate the required computational overhead of the five methods to complete an optimal SVM model, we have recorded the computational time in seconds of using the five methods to implement the training and prediction procedure in the fold #4 run of the GCD dataset, and these results are presented in Fig 15. This graph shows that the proposed CIFOA-SVM requires an average of almost 800 seconds to implement the training and prediction procedure in the fold #4 run for the GCD dataset. Compared with CIFOA-SVM and GAFS on the GCD dataset, the required running time was reduced by 40 seconds by CIFOA-SVM. Compared with the PSOFS and CIFOA-SVM, the required running time was reduced by 215 seconds by CIFOA-SVM. Compared with CIFOA-SVM and TVPSOFS, the running time was reduced by 35 seconds by CIFOA-SVM. Moreover, the same phenomenon has been observed in Fig 15. PSOFS requires the most time consumption for implementing the entire optimization process.

**Fig 16:**
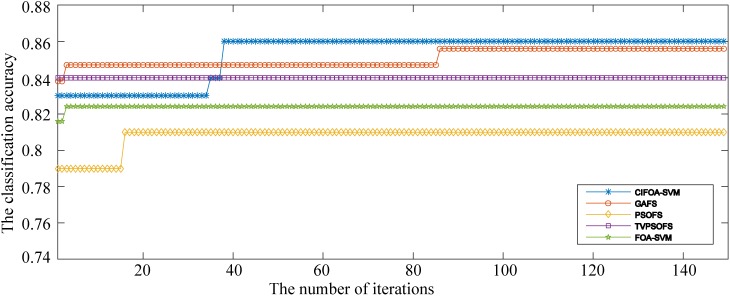
The convergence curves generated for fold #5 run by the five methods for German Credit Data (GCD).

To compare the searching capability of using various intelligent algorithms to determine the optimal parameter setting of the SVM model and to select a proper feature subset, this experiment recorded the best classification accuracy achieved by the five methods in each iteration for fold #4 of the GCD dataset. Fig 16 shows the convergence curve generated by the five methods with increasing iterative times for the GCD dataset. This curve shows that did the proposed CIFOA-SVM not only almost achieve the best classification accuracy of all the methods for different iterations but also determined the superior solution and met the termination criteria far more rapidly.

## 5. Conclusion and future work

In this work, feature selection and parameter estimation for the SVM are transformed into a complex multidimensional optimization problem. To solve this problem and obtain an optimal SVM model, this study proposed an improved FOA based on the chaotic PSO–in combination with the mutation strategy. In the proposed method, the proposed improved algorithm, CIFOA, has been successfully applied to determine the optimal parameter setting of the SVM classifier and to provide a more appropriate feature subset. To prevent the searching procedure from becoming trapped in a local optimum and to have an efficient classifier with better global searching ability, a mutation strategy was proposed to maintain population diversity. In this study, we first perform several groups of tests to evaluate the searching ability of the proposed CIFOA in solving the complex nonlinear continuous functions. The empirical results show that CIFOA not only achieves a significant result with respect to parameter estimation of the optimization function but also has a faster convergence rate. Finally, to evaluate the effectiveness of parameter estimation of the proposed improved algorithm and the classification performance of the proposed intelligent framework, CIFOA-SVM, we performed several groups of experiments using various well-known methods for solving the credit card problem and the medical diagnosis problem. The experimental results reveal that the proposed intelligent framework is a powerful tool for parameter optimization and feature selection for SVM.

There are several notable directions for our future work. First, it would be interesting to combine the proposed intelligent framework with a different classifier–such as a Naïve Bayes and an Artificial Neural Network–to solve classification problems in wider areas. Second, we would like to extend the proposed intelligent framework to solve multi-class problems in the real world. Finally, it would be fruitful to employ heterogeneous evolution algorithms with the swarm optimization technique to construct several groups of experiments for classification tasks.
